# Using movement to inform conservation corridor design for Mojave desert tortoise

**DOI:** 10.1186/s40462-020-00224-8

**Published:** 2020-10-06

**Authors:** Steven J. Hromada, Todd C. Esque, Amy G. Vandergast, Kirsten E. Dutcher, Corey I. Mitchell, Miranda E. Gray, Tony Chang, Brett G. Dickson, Kenneth E. Nussear

**Affiliations:** 1 Program in Ecology, Evolution and Conservation Biology, University of Nevada, 1664 N. Virginia St, Reno, NV 89557 USA; 2 Department of Geography, University of Nevada, 1664 N. Virginia St, Reno, NV 89557 USA; 3 U.S. Geological Survey, Western Ecological Research Center, 160 N Stephanie St, Henderson, NV 89074 USA; 4 U.S. Geological Survey, Western Ecological Research Center, 4165 Spruance Road Suite 200, San Diego, CA 92101 USA; 5 Conservation Science Partners, 11050 Pioneer Trail, Suite 202, Truckee, CA 96161 USA; 6 Landscape Conservation Initiative, Northern Arizona University, P.O. Box 5694, Flagstaff, AZ 86011 USA

**Keywords:** Connectivity, Path-selection, Home range, Utility-scale solar

## Abstract

**Background:**

Preserving corridors for movement and gene flow among populations can assist in the recovery of threatened and endangered species. As human activity continues to fragment habitats, characterizing natural corridors is important in establishing and maintaining connectivity corridors within the anthropogenic development matrix. The Mojave desert tortoise (*Gopherus agassizii*) is a threatened species occupying a variety of habitats in the Mojave and Colorado Deserts. Desert tortoises have been referred to as corridor-dwellers, and understanding how they move within suitable habitat can be crucial to defining corridors that will sustain sufficient gene flow to maintain connections among populations amidst the increases in human development.

**Methods:**

To elucidate how tortoises traverse available habitat and interact with potentially inhospitable terrain and human infrastructure, we used GPS dataloggers to document fine-scale movement of individuals and estimate home ranges at ten study sites along the California/Nevada border. Our sites encompass a variety of habitats, including mountain passes that serve as important natural corridors connecting neighboring valleys, and are impacted by a variety of linear anthropogenic features. We used path selection functions to quantify tortoise movements and develop resistance surfaces based on landscape characteristics including natural features, anthropogenic alterations, and estimated home ranges with autocorrelated kernel density methods. Using the best supported path selection models and estimated home ranges, we determined characteristics of known natural corridors and compared them to mitigation corridors (remnant habitat patches) that have been integrated into land management decisions in the Ivanpah Valley.

**Results:**

Tortoises avoided areas of high slope and low perennial vegetation cover, avoided moving near low-density roads, and traveled along linear barriers (fences and flood control berms).

**Conclusions:**

We found that mitigation corridors designated between solar facilities should be wide enough to retain home ranges and maintain function. Differences in home range size and movement resistance between our two natural mountain pass corridors align with differences in genetic connectivity, suggesting that not all natural corridors provide the same functionality. Furthermore, creation of mitigation corridors with fences may have unintended consequences and may function differently than natural corridors. Understanding characteristics of corridors with different functionality will help future managers ensure that connectivity is maintained among Mojave desert tortoise populations.

## Introduction

Maintaining genetic connectivity between animal populations is a crucial step in ensuring the long-term viability of species [1–4]. Typically, conservation plans incorporating connectivity goals call for the establishment of corridors—regions of the landscape that serve to maintain or facilitate functional connections between populations of organisms through areas of inhospitable landscape [5–7]. Depending on the size and configuration of the inhospitable landscape and movement capabilities, different species may have different needs from corridors intended to preserve historic genetic connectivity—some may need suitable dispersal habitat for short term movements (i.e. corridor-passers), while other species need to reside within the corridor, requiring a corridor that can allow for population residency and transgenerational connectivity (i.e. corridor-dwellers) [8]. As human development continues to encroach into natural areas, there is interest in, and a clear need for, properly designed corridors that can link wildlife populations through areas dominated or altered by human activity [4, 9]. Maintaining connectivity across the landscape of the desert southwest of the United States has become an increasing concern as landscape-level disturbances (utility-scale renewable energy, urbanization, transportation infrastructure, military training, and vehicle recreation areas) have been and continue to be sited within relatively intact natural landscapes [10–12].

Characterizing animal movement, which directly links a species’ ecology to connectivity, has been considered one of the optimal methods for describing functional or dysfunctional corridors [9, 13, 14]. The advent of miniaturized GPS dataloggers has led to a wealth of data that can better characterize fine-scale animal movement through the landscape [15]. Statistical methods (such as path-selection or step-selection) that use movement data have been shown to outperform prior connectivity quantifications based on methods such as species distribution models and expert opinion [13]. However, these methods have predominantly been applied to large-bodied, vagile species that would be considered corridor-passers [16–18]. Although recommendations exist for corridors designed for corridor-dwelling species [8], few studies have used movement data to inform corridor design for corridor-dwellers, instead relying on genetic data, mark-recapture surveys, or species distribution models [19–21]. Movement and habitat quality requirements can vary among species. Corridors that contain suitable habitat yet contain low quality movement habitat may alter patterns of historic gene flow across a landscape; thus, understanding metrics that predict movement quality for a species can be important [6]. Understanding these differences can be especially important for corridors adjacent to human development, where the anthropogenic edges of the corridor may alter animal behavior and resultant corridor functionality [22]. The primary prescription of corridor design for corridor-dwellers is the ability to fit multiple overlapping home ranges within the corridor, yet home range sizes may not necessarily be consistent, particularly for species with wide distributions across heterogeneous environments [23, 24].

The Mojave desert tortoise (*Gopherus agassizii*) is a terrestrial, herbivorous reptile native to the Mojave and Colorado Deserts of the southwestern United States. The majority of Mojave desert tortoise habitat falls in bajadas (coalescing alluvial fans) and valleys, though some populations exist in rugged mountainous terrain, and mountain passes between neighboring valleys may serve as important natural corridors between major populations [25, 26]. Although listed as a threatened species under the United States Endangered Species Act in 1990, population size and the range of the tortoise continues to rapidly decline due to many threats including land development, disease, livestock grazing, subsidized predators, off-highway vehicle recreation, wildfire, and invasive plants [27–30]. Studies on the genetic connectivity of the species indicate a general pattern of range-wide isolation-by-distance with topographic features such as mountain ranges and deep valleys serving as barriers to gene flow [31, 32]. Maintaining historic range-wide connectivity has been identified as a key factor in the recovery of the species, thus understanding how new anthropogenic features may influence tortoise movement is critical to land management decisions [28, 33]. The low mobility of this species has led to its classification as a corridor-dweller [33], and a pressing concern is the configuration of functional corridors to maintain historic connectivity around new landscape-level disturbances within formerly contiguous habitat. The original 1994 recovery plan for the Mojave desert tortoise recommended that “Connecting habitat segments should be of medium to high quality and be wide enough to accommodate several desert tortoise home-range widths (several miles)” be used as corridors connecting smaller patches of preserved tortoise habitat [34]. The updated 2011 recovery plan offered no new recommendations on corridor design other than to conduct applied research to “Determine the importance of corridors and physical barriers to desert tortoise distribution and gene flow” [28]. More recently, consideration of the connectivity of all existing habitat, instead of just between protected areas has been emphasized [33].

Much of desert tortoise habitat has been intersected by transportation infrastructure, ranging from single track recreation trails to superhighways and railroads [30]. Tortoises are particularly susceptible to road impacts; their slow speed makes them likely to be hit by vehicles, and roads often serve as conduits for predators and invasive plants [27, 35–37]. As road mortality has been identified as an important factor in the decline of the desert tortoise, roadside tortoise exclusion fences have been constructed along many major highways in the Mojave Desert [38, 39]. Additionally, exclusion fences are typically erected around utility-scale energy infrastructure to prevent tortoises from returning to former home-ranges post-translocation. Research on the closely related gopher tortoise (*Gopherus polyphemus*) suggests that railroads may also serve as nearly impassable barriers to movement for tortoises [40]. Understanding how these disturbances affect desert tortoise movement and space use is critical in siting future anthropogenic development and designing and maintaining effective corridors within existing tortoise habitats.

To characterize natural and mitigation corridors for tortoise population connectivity, we describe a model of tortoise movement generated from fine-scale GPS telemetry data that indicates path selection in response to both natural features and anthropogenic disturbances. This model was informed by the fine-scale movement of tortoises within both open and movement-limiting habitat (rugged terrain), as well as areas with common anthropogenic disturbances. We predict landscape-scale resistance and use this to describe differences between two natural corridors through mountain passes, and existing mitigation corridors in Ivanpah Valley, which are mitigation for utility-scale solar installations. As home range size is often important in recommendations for corridor-dwelling species, we calculate and examine differences in home range size of tortoises living in these corridors and open valley areas. By describing how movement and home-ranges differ between natural and anthropologically altered corridors, we provide insight on how to maintain connectivity of tortoise populations.

## Methods

### Study area and tortoise telemetry

**Fig. 1:**
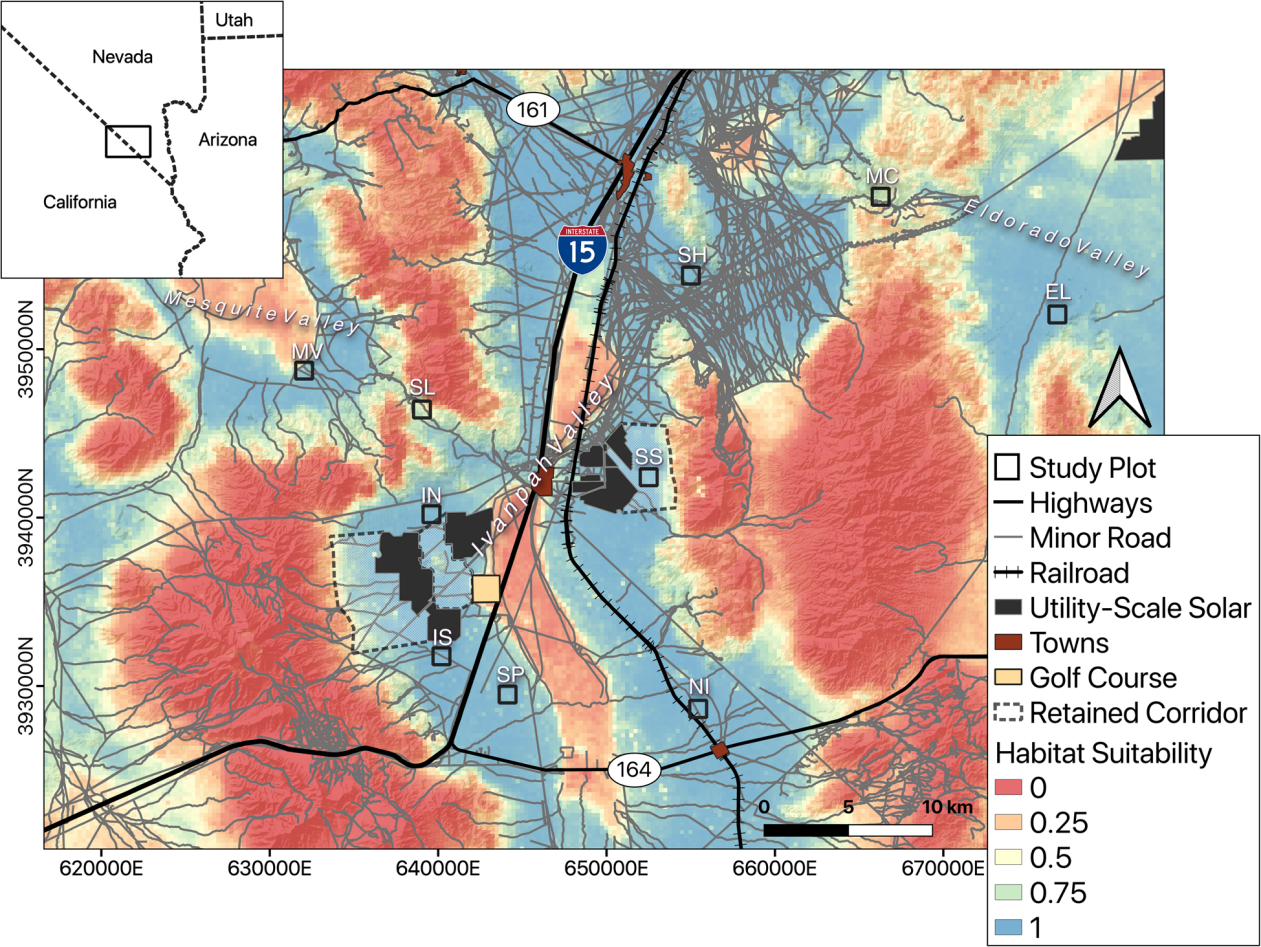
Map of study area in the Ivanpah Valley area on the Nevada/California border, USA. Shown are study plots, anthropogenic disturbances (urban areas, utility scale solar, golf course), transportation infrastructure (highways, minor roads, railroads), and mitigation corridors (white hatched areas) overlaid on a Mojave desert tortoise habitat suitability model from Nussear and Simandle 2020. Site codes (White text above study plots): MC (McCullough Pass), SH (Sheep Mountain), SS (Silverstate), SL (Stateline Pass), IN (ISEGS North), MV (Mesquite Valley), NI (Nipton), EL (Eldorado Valley), IS (ISEGS South), SP (Southpah)

We studied tortoise movements in and adjacent to the Ivanpah Valley, on the border of Nevada and California, which is an important area for range-wide connectivity of the species [33, 41]. Tortoise habitat within the Ivanpah Valley has been subject to habitat alteration for over a century, and contains myriad different anthropogenic features, including paved and unpaved roads, several small urban areas, many abandoned and active mines, Interstate 15, railroads, a golf course, and, in the past decade, three utility scale solar energy facilities (Ivanpah Solar Energy Generating System [ISEGS], Stateline Solar Farm, and Silver State South Solar Energy Center; Fig. 1). The solar facilities were sited in previously undeveloped desert tortoise habitat, with estimated pre-construction adult tortoise densities between 1.2–10.4 tortoises/km^2^ [42–45]. Areas of suitable tortoise habitat were retained to act as corridors between each solar facility and modeled unsuitable habitat, intended by the United States Bureau of Land Management (BLM) to mitigate for the loss of roughly 3275 ha of high-quality tortoise habitat (Fig. 1). These corridors provide an area about two kilometers wide; narrower areas of suitable tortoise habitat also exist within and between the different solar facilities. The intention of these mitigation corridors was to conserve genetic and demographic connectivity of tortoise populations throughout the Ivanpah Valley [42–45].

We established ten 1-km^2^ plots in areas of suitable desert tortoise habitat within land managed by the BLM [25]: six plots were within Ivanpah Valley, one plot each in the neighboring Eldorado and Mesquite valleys, and two plots in mountain passes connecting the valleys (Fig. 1). Vegetation at the sites is Mojave Desert scrub with a *Larrea tridenta*-*Ambrosia dumosa* association; most sites also contain *Yucca schidigera*, and a small portion of the site in Mesquite Valley includes *Prosopis pubescens* thickets. Soil textures vary among sites—soil in the lower elevations of Ivanpah Valley is typically sandy, with increasing rock and cobble at higher elevations; the mountainous sites are generally rocky and rugged.

Seven of our ten plots contain dirt roads; the BLM classifies these open routes as “improved” and suitable for 4WD and ATV use, although non-designated trails exist as well. All plots have a road within 1 km of the plot boundary. One site (Nipton) contains a low-traffic paved road that exists as a service road to the parallel railroad, which is also partially within the plot. The railroad is raised approximately three meters above ground level, and several peripheral flood control structures (raised berms) were constructed to divert water into underpasses beneath the tracks. The flood control berms are approximately 300–700 m long, form an angle with the ground at about 35°, and are mostly constructed of soil about 2 m high, though the section (~ 150 m long) closest to the railroad is concrete. Three of the plots are located adjacent to the recent utility-scale solar energy installations; each installation is fenced to exclude terrestrial wildlife, including tortoises which were translocated prior to construction [45, 46].

Both resident and translocated tortoises were tracked between 2016 and 2018. Tortoises were incorporated into the telemetry study in several different ways: tortoises were found during preliminary surveys in the areas around the Stateline Pass and McCullough Pass plots in 2012; plots were systematically surveyed for tortoises from 2015 to 2018 with visual encounter surveys; and tortoises were subsequently encountered opportunistically during telemetry activities. Additionally, some tortoises inhabiting the vicinity of plots were transferred from other projects– e.g., tortoises that were part of government-mandated monitoring efforts related to utility-scale solar construction. These tortoises were already equipped with VHF (Very High Frequency) transmitters and included residents known to occur within our study plots, as well as tortoises that were translocated out of the footprint of utility-scale solar energy facilities. Translocations occurred in 2012 for the ISEGS facility (1 tortoise used in this study) and 2014 for the Silver State Solar facility (13 tortoises used in this study). Based on prior research into the effects of translocation on Mojave desert tortoises, we did not anticipate that translocated tortoises would have different habitat selection or movement patterns after 2 years post translocation [47, 48]. Resident tortoise populations on our plots show an overall pattern of genetic isolation-by-distance and were classified into three geographically distinct genetic clusters that also indicated moderate genetic mixing resulting from gene flow: Ivanpah/Mesquite valleys, Eldorado Valley, and McCullough Pass [26].

All adult tortoises (generally MCL [Midline carapace length] > 200 mm) in the project were fitted with a VHF radio transmitter (Holohil Systems Ltd., Ontario, Canada) and a custom build sled to hold a GPS logger (i-gotU GT-120). Equipment weighed less than 5% of each individual’s mass and was installed to minimize their vertical profile on the animal. Tortoises were relocated once a month, and GPS loggers were retrieved and swapped during tracking encounters when the animal was available for handling (generally April–October). GPS loggers were programmed to record points hourly, though rough terrain and burrow use often temporarily precluded point collection.

### Environmental layers

Based on prior research on tortoise movements in the Ivanpah Valley [49], we anticipated that natural landscape features would influence tortoise movement selection. We derived topographical covariates from the 30-m USGS Digital Elevation Model [50]. Slope is an important factor in tortoise habitat suitability as well as movement ability [51, 52]. Desert washes and ephemeral streambeds are prominent landscape features in the Mojave Desert and are important in desert tortoise ecology, both as foraging areas and potentially as movement corridors [53, 54]. We used a wash layer derived using USDA National Agricultural Imagery Program (NAIP) [55] and classified pixels by the “intensity of wash characteristics” using a random forest model at a 1-m resolution (further details in [48, 52]). Washes typically have different substrates and vegetative communities than the surrounding uplands; pixels scored as high wash intensity in the model had lower vegetation cover.

Perennial shrubs are important for desert tortoises as thermal refugia and provide integrity for burrow construction [51, 56, 57]. To approximate shrub availability, we used a vegetation layer derived from early summer 2016–2017 NAIP NDVI (normalized difference vegetation index) imagery, to capture the variability in perennial shrub cover within and between our sites while eliminating annual variation in spring and late-summer annual plant growth (further described in [52]).

To understand if general habitat suitability influences tortoise movement, we used a habitat model developed for Mojave desert tortoises for the Clark County Desert Conservation Program [58]. This model was developed using tortoise presence locations and climatic and soil characteristics at a 250-m resolution within Clark County, Nevada and a surrounding 50 km buffer. The model used an ensemble method that incorporated the results of three different modeling algorithms (Random Forest, MaxEnt, and Generalized Additive Models) using the biomod2 package in R (v 3.3–7.1 [59]). Models were assessed with cross-validation runs using common model performance metrics (AUC [Area Under the Curve], TSS [True Skill Statistic]) and the final model was produced by averaging model predictions across 50 cross-validation runs (Fig. 1, further modeling details in [58]).

### Human disturbances

We created rasters representing distance to human disturbances to understand how human disturbances on the landscape influence tortoise habitat selection. All roads, railroads, fences and flood control structures were digitized from aerial imagery [60]. A raster was then created that represented the distance to the nearest human disturbance from focal tortoises using the gDistance function from the gDistance package v. 1.2–2 [61] in program R v. 3.5.3 [62]. As all the roads in our study plot have low traffic, we considered them as one covariate in analyses. We took a natural log of the distance and “flattened” the rasters above a value representing 60 m. This preserved the short-range effect of these disturbances, to allow inference on the localized effect that these disturbances create on the landscape [63]. We acknowledge that this may not be the exact range that tortoises perceive anthropogenic disturbances, but this distance represents a clear break-point in the values of our rasters and was a better fit in preliminary modeling than using a non-flattened raster [36, 39]. Separate rasters were created in the same manner to represent the localized “distance to fence”, “distance to railroad”, and “distance to flood control berms” for the three sites that are located adjacent to utility-scale solar exclusion fencing and the one site containing the railroad and associated flood control berms.

### Analysis

#### GPS data cleaning

Erroneous tortoise GPS locations were identified and removed from the dataset using several criteria: an estimated elevation error (the difference between the recorded elevation and elevation from the USGS DEM) above 25 m (based on the error distribution in [64]), points that would have required the tortoise to move faster than they are able to (300 m/hour; Nussear and Esque, unpublished data), and points with a high speed (> 100 m/hr) and a sharp turning angle (> 300°) from the prior and to the next point locations. Additionally, our choice to use a 30-m resolution for covariate rasters was intended to account for potential locational error from the retained GPS points, which we expected to be < 10 m [65]. GPS data were only used from years in which we had an entire active season (spring through fall) of tracking at a site.

#### Home-range estimation

Annual home-ranges for each tortoise were estimated using an auto-correlated kernel density method in the R package ctmm (v0.5.9 [66]). This method fits the telemetry data (both from VHF tracking and GPS loggers) with a continuous time stochastic process movement model to fit an appropriate kernel bandwidth for each individual tortoise to account for spatiotemporal autocorrelation inherent in fine-scale GPS logger datasets and properly estimate home ranges. As tortoise home-ranges can change annually based on environmental conditions [67, 68], we estimated annual home ranges for each tortoise with more than 5 months of GPS logger data per active season (April–October; 2015–2018 [69]). To generate the home-range areas for tortoises that encountered fences, we removed areas of estimated home-range that fell within fenced areas. We tested for differences in home-range between sites and between tortoises that encountered fences and those that didn’t using a mixed effects linear model with site, sex (including juvenile as a sex for tortoises not mature enough to display secondary sex characteristics [e.g. enlarged gular, plastron concavity]), year and fence as covariates. Analysis was done in R package lme4 v. 1.1–21 [70], with *p*-values calculated using Satterthwaite’s method in R package lmerTest v. 3.1–2 [71].

### Path selection analysis

#### Path Construction

We subsetted the entire tortoise GPS point dataset using functions provided in package adehabitatLT (v 0.3.24 [72]) to represent tortoise movement paths through the landscape by applying the following criteria: paths had to have three or more points, points had to be from fixes an hour apart, the distance between each set of points had to be greater than 10 m (the expected error of our GPS units [65]), and the total distance moved had to be greater than 100 m.

**Fig. 2:**
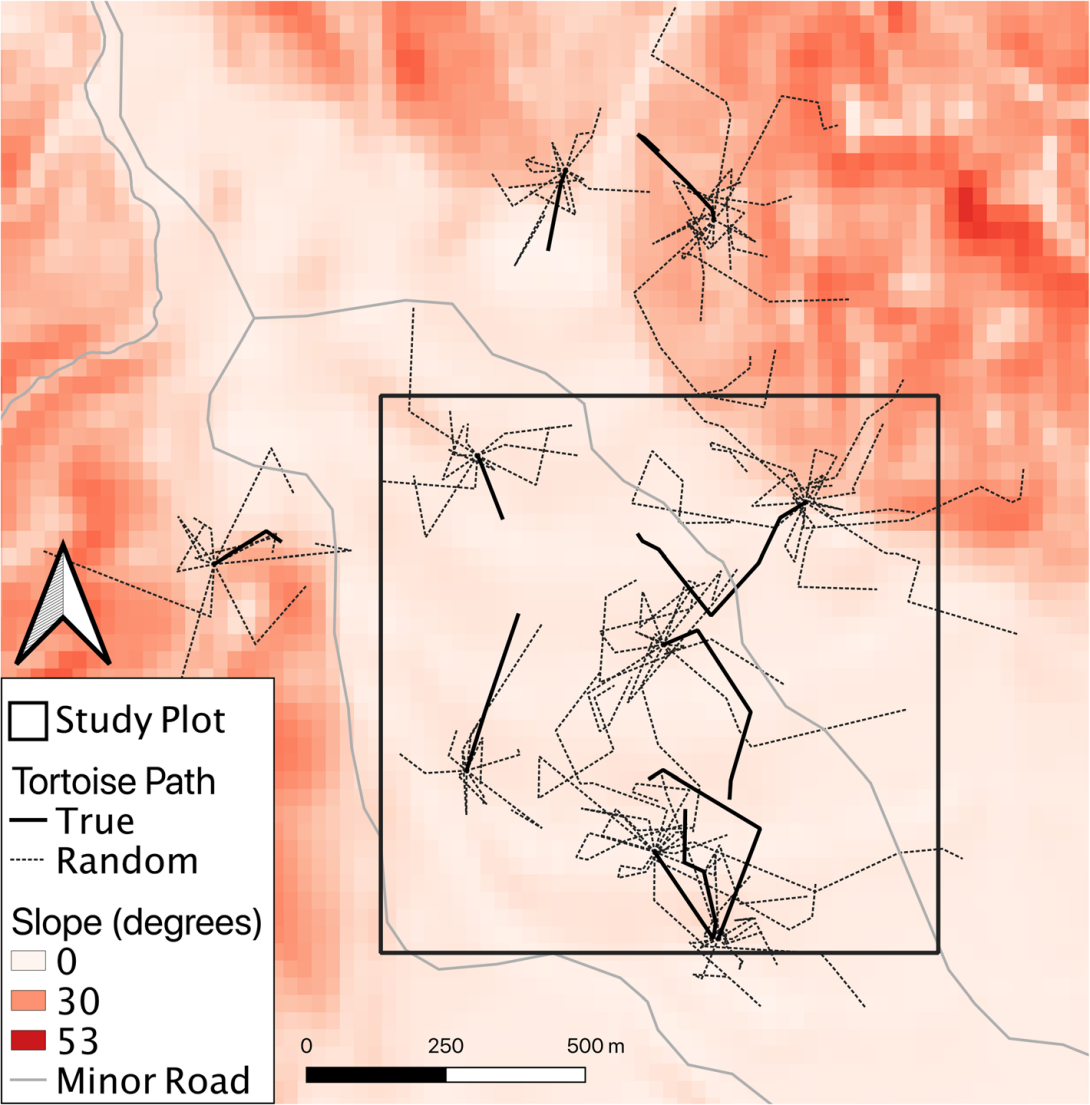
Example of true and random tortoise paths in the Stateline Pass study plot, San Bernardino County, CA. For each true tortoise path ten random correlated walks were generated and overlaid on covariates

We then created 10 random paths for each real tortoise movement path, starting at the location of the true path and progressing for the same number of steps (Fig. 2). Step lengths and turning angles were drawn from distributions (gamma for step lengths, wrapped normal for turning angle) fitted from the entire true path dataset, to avoid issues of circularity that could arise from solely using the distribution of an individual’s step parameters [73, 74]. We then extracted the mean of all the raster values of each covariate along each true and random path, creating a “cost” of movement for each path. Using full-length random paths allowed us to sample “available” habitat farther away from tortoise activity centers that would potentially be unsampled by other methods of quantification (e.g. Brownian bridges, step-selection [75, 76]). Path selection produces more robust selection parameter estimates than step-selection functions, and longer paths through the landscape are likely to be the most meaningful resolution for hourly data [77].

**Model fitting**

Individual animals can differ in habitat selection preferences; accounting for these potential differences and differences in habitat availability is important to allow a model to account for these differences among study animals and reduce false precision in regression coefficients [78–80]. To compare the used path to the random paths while allowing for individual-level variability in selection coefficients, we implemented a framework similar to that described in [81]. This framework exploits the fact that a conditional logistic model is the likelihood equivalent to a Poisson-model with stratum specific-fixed intercepts, allowing estimation of selection using random coefficients for each individual study animal. We fit models using different subsets of variables, and compared models with WAIC (Widely Applicable Information Criterion) values from progressively more complex fitted models using integrated nested Laplace approximation (INLA) to find the model with the lowest WAIC value (INLA v. 19.09.03 [82, 83]).

As we anticipated that some selection responses may be non-linear, we compared models fit with both a linear and non-linear predictor (e.g. slope vs slope^2^) and chose which parameterization provided a lower WAIC score. We also fit models using interaction terms between natural features and interaction terms between natural covariates (slope and wash) and road distance, as many roads in our plots were located within washes, and tortoises may respond differently to roads in areas of high slope.

We defined whether a tortoise encountered an anthropogenic feature by looking for intersections between the feature and 95% Maximum Likelihood autocorrelated home range estimates for each tortoise. Not all tortoises encountered anthropogenic disturbances—rather than subsetting out tortoises based on the features with which they encountered, we gave NA values to path values for anthropogenic features for tortoises that did not encounter that feature. INLA will not attempt to fit covariates for an observation with NA values, allowing us to use data from tortoises that encountered anthropogenic features alongside tortoises that never encountered anthropogenic features in the same model.

To understand the spatial distribution of tortoise movement habitat within the broader landscape around our study plots, we used the coefficients from our best model to predict movement habitat quality across rasters of predictors in the greater Ivanpah Valley area. In order to avoid having movement quality predictions confounded by modeled selection for linear barriers (the highest quality movement habitat was predicted along fences), the values for distance to fence, flood control and railroad were held constant. These values were put into “fisher” breaks categories using the classInt package in R (v 0.4–2, [84]) to aid in visualization. We overlaid least-cost paths from [31] onto our map of movement quality predictions to compare with prior research into range-wide genetic connectivity for the tortoise. These least-cost paths were based on a resistance surface derived from a tortoise habitat suitability model, and were found to be the best supported model for describing range-wide genetic distances [31].

## Results

We tracked 129 tortoises with GPS loggers over a time span of 3 years, resulting in 586,911 GPS locations (Table 1).

**Table 1:** Summary of ten study sites, telemetered tortoises with GPS loggers, and years with a full active season (Spring-Fall) of GPS logger data at ten sites in and around the Ivanpah Valley from 2016 to 2018

Site	Tortoises	Years	GPS points
McCullough Pass (MC)	26	2016–2018	112,180
Sheep Mountain (SH)	12	2016–2018	102,263
Silver State (SS)	15	2016–2018	110,498
Stateline Pass (SL)	7	2017–2018	15,472
ISEGS North (IN)	9	2017–2018	47,579
Mesquite Valley (MV)	6	2017–2018	20,952
Nipton (NI)	15	2017–2018	53,249
Eldorado (EL)	10	2018	17,032
ISEGS South (IS)	6	2018	17,002
Southpah (SP)	24	2018	90,684
Total	130		586,911

### Home range estimation

**Fig. 3:**
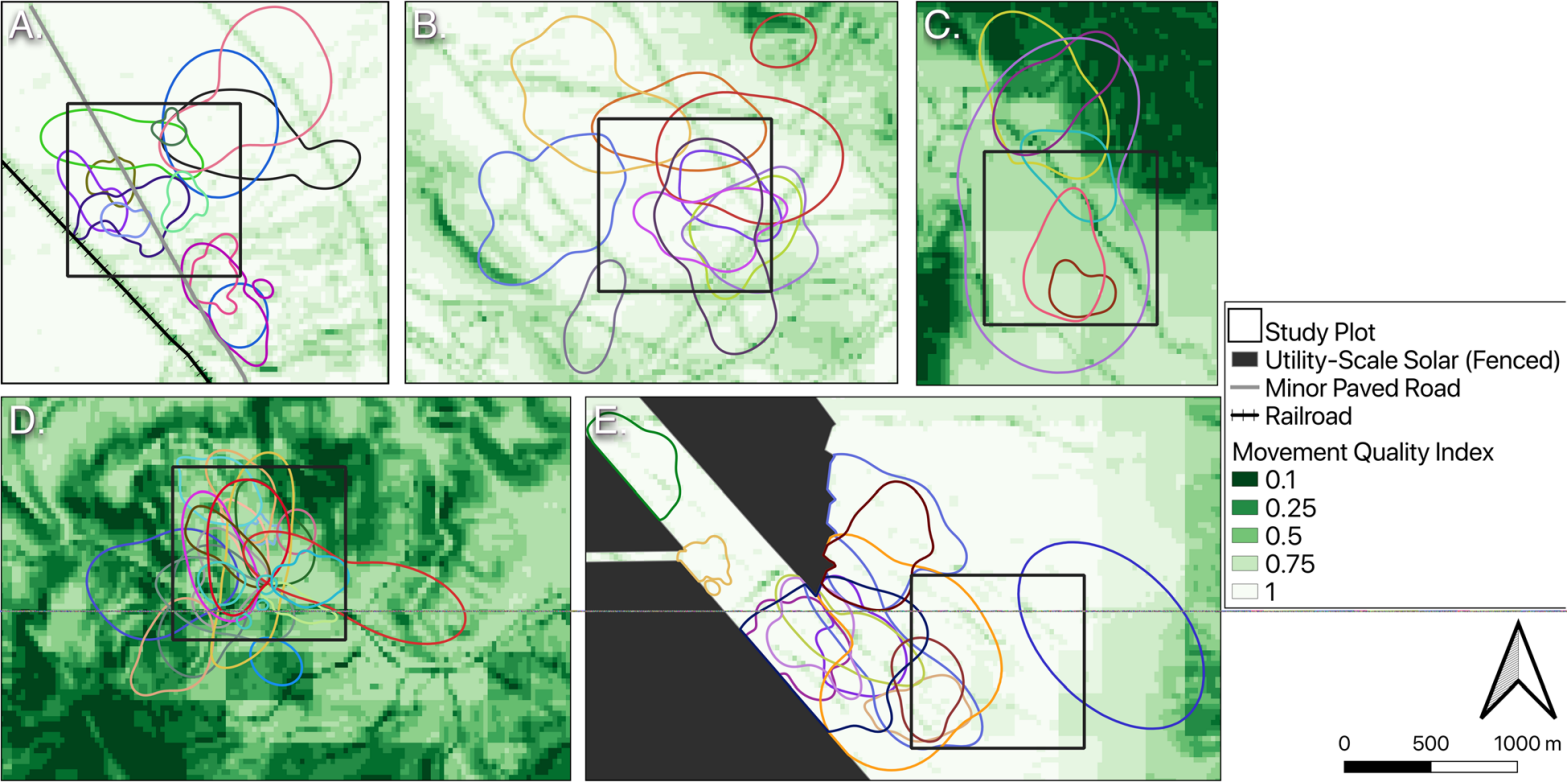
Estimated annual home ranges for desert tortoises at different study plots in the Ivanpah Valley area, NV/CA. Each color polygon represents an individual tortoise that was tracked during the entire active season (April–October) of 2018. Home ranges of tortoises in the Nipton plot (**a**) often had boundaries corresponding to the paved road, while the same was not generally true for dirt roads in the Sheep Mountain plot (**b**). Home ranges of tortoises in the mountain pass plots often included areas of lower movement quality (**c** & **d**). Fences formed boundaries of home ranges of tortoises living near utility-scale solar fields (**e**)

We estimated home ranges for 32 tortoises in 2016, 44 tortoises in 2017, and 105 tortoises in 2018. Of these tortoises, three in 2016, seven in 2017, 11 encountered fences in 2018. The movement models fit to GPS data indicated that spatiotemporal autocorrelation in the relocation data was roughly 14 days for our tortoises. Most estimated annual home ranges had areas from 2.2 ha to 100 ha, with several over 100 ha (two in 2016, seven in 2017, and ten in 2018); as expected an individual tortoise’s home range often varied among years, though year was not overall a significant factor likely due to lack of replication across years for many sites (ANOVA: F_2,159.4_ = 1.53, *P* = 0.22; Table 2). Home-range size varied among plots and between tortoise sexes (ANOVA: F_9,106.6_ = 3.42, *P* = 0.001; ANOVA: F_2,113.9_ = 11.7, *P* < 0.001). Tortoises in the McCullough Pass plot tended to have smaller home ranges than tortoises in other plots, with the exception of the Nipton plot which contains the railroad and a paved road (Table 2). Tortoises that encountered fences did not have smaller home ranges than tortoises that never encountered the fences within a year (ANOVA: F_1,91.48_ = 0.03, *P* = 0.85; Table 2). Home ranges for six of the ten tortoises that lived near the paved road in the Nipton plot had boundaries roughly corresponding with the edge of the paved road (Fig. 3a), while tortoises living near dirt roads generally did not show the same relationship (e.g. Sheep Mountain plot (Fig. 3b)). Fences formed the edges of estimated home ranges for tortoises that lived near them (Silver State plot (Fig. 3e)). Estimated home ranges of tortoises living on the mountain pass plots generally contained areas of lower predicted movement quality (Fig. 3c-d).

**Table 2:** Summary of estimated home range sizes (in ha) for adult Mojave desert tortoises in the greater Ivanpah Valley Area. Estimates were made using autocorrelated kernel density estimators in R package ctmm. Tortoises that encountered fences are included in a separate row. Minimum and maximum values are not displayed when only one tortoise from a site had sufficient data for that year

Site	2016			2017			2018		
	Min	Median	Max	Min	Median	Max	Min	Median	Max
McCullough Pass	4.03	**17.56**	259.43	2.95	**12.53**	100.76	2.20	**14.15**	43.26
Sheep Mountain	21.43	**50.50**	190.35	18.05	**30.49**	250.43	21.28	**46.64**	355.86
Silver State	17.39	**21.90**	54.63	13.36	**24.50**	187.36	15.78	**24.0**	102.49
Silver State (fence)	25.85	**35.85**	44.31	3.12	**35.40**	46.25	6.05	**28.62**	100.23
Stateline Pass				13.40	**46.86**	102.31	25.13	**39.83**	169.01
ISEGS North				27.97	**43.64**	52.51	16.12	**55.09**	111.79
Mesquite Valley				20.32	**151.27**	184.83	44.83	**117.78**	230.61
Nipton					**17.26**		5.23	**11.95**	55.12
Eldorado Valley							10.05	**35.22**	409.80
ISEGS South							17.23	**38.54**	91.00
ISEGS South (fence)								**51.17**	
Southpah							3.73	**29.99**	200.07

### Path Selection

Using these location data, we created a total of 4053 paths representing movements of the 101 tortoises that had more than 5 paths. Based on intersection of a feature with home ranges, 62 tortoises encountered roads, 16 tortoises encountered fences, 7 encountered railway infrastructure (including flood control berms), and only 2 of those 7 directly encountered the railroad itself.

Models fitted with covariates accounting for distance to roads, fences, and flood control berms performed better than the null model (Table 3). The addition of distance to the railroad did not improve WAIC score by itself, but models with both the railroad and associated flood control covariates together were ranked higher than models with either one alone. For this reason and to infer how tortoises interact with the railroad, we retained all four human disturbance covariates.

After sequentially adding the natural covariates to models with human disturbance covariates, our three highest ranked models were within two WAIC of each other (Table 3). We chose to proceed with the model that did not contain the quadratic term for vegetation and the road/wash interaction, as posteriors for both these covariates were centered around zero, implying little explanatory power.

**Table 3:** Model selection table for desert tortoise path selection ranked by WAIC. The top three models were highly similar in WAIC score (in bold)

Model	WAIC	ΔWAIC
**slope + slope**^**2**^ **+ wash+wash**^**2**^ **+ veg + veg**^**2**^ **+ habitat+road+fence+floodcontrol+rail**	35090.50	0.00
**slope + slope**^**2**^ **+ wash+wash**^**2**^ **+ veg + veg**^**2**^ + habitat+road+fence+floodcontrol+rail+road*wash	35090.56	0.06
**slope + slope**^**2**^ **+ wash+wash**^**2**^ **+ veg + habitat+road+fence+floodcontrol+rail**	35092.14	1.64
wash+wash^2^ + habitat+road+fence+floodcontrol+rail	35131.25	40.75
wash+habitat+road+fence+floodcontrol+rail	35159.45	68.95
slope + slope^2^ + wash+ wash^2^ + veg + veg^2^ + habitat road+fence+floodcontrol+rail+slope*wash	35182.47	91.97
slope + slope^2^ + habitat+road+fence+floodcontrol+rail	35184.42	93.92
road+slope + fence+floodcontrol+rail+habitat	35188.02	97.52
veg + veg^2^ + habitat+road+fence+floodcontrol+rail	35214.96	124.47
road+veg + fence+floodcontrol+rail+habitat	35221.50	131.00
habitat+road+fence+floodcontrol+rail	35228.94	138.44
habitat+road+fence+floodcontrol	35233.82	143.33
habitat+road	35281.80	191.31
habitat+floodcontrol+rail	35310.44	219.95
habitat+floodcontrol	35311.56	221.07
habitat+fence	35330.54	240.05
habitat	35341.03	250.54
habitat+rail	35342.31	251.82
Null	35368.26	277.77

**Fig. 4:**
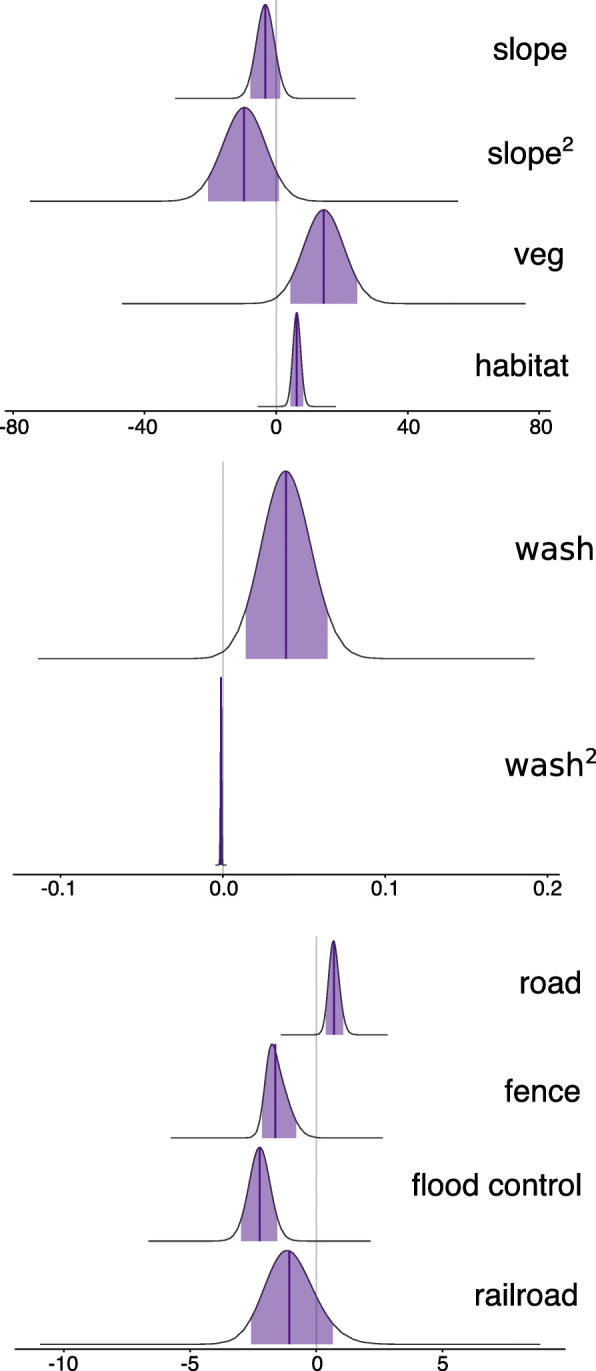
Posterior estimates for selection coefficients from path selection analysis on Mojave desert tortoises. Results for natural terrain covariates indicate that tortoises exhibit selection against high levels of slope, and selection for higher habitat suitability, perennial vegetation cover, and low but not high levels of wash characteristics. Coefficients for human disturbance features suggest that tortoises select to move farther distances from roads, while selecting to move along impassible linear barriers (fences, flood control berms, railroad)

Coefficients for the best model indicate that tortoises make movement choices based on natural features; our posteriors indicate a reluctance to move through areas of high slope, and a preference for higher vegetation cover and wash characteristics (Fig. 4). The negative values for the posteriors of the quadratic terms imply that tortoises will select against slope and for higher wash characteristics until a certain cutoff value where the selection asymptotes or reverses. We found almost no probability of movement at about 30° for slope, a maximum probability of movement at around 20 units of wash characteristics, and a selection against movement at wash characteristic values above 40. These values of wash characteristics correspond with the typical washes found in tortoise habitat; higher values represent more incised washes with less vegetative cover.

**Fig. 5:**
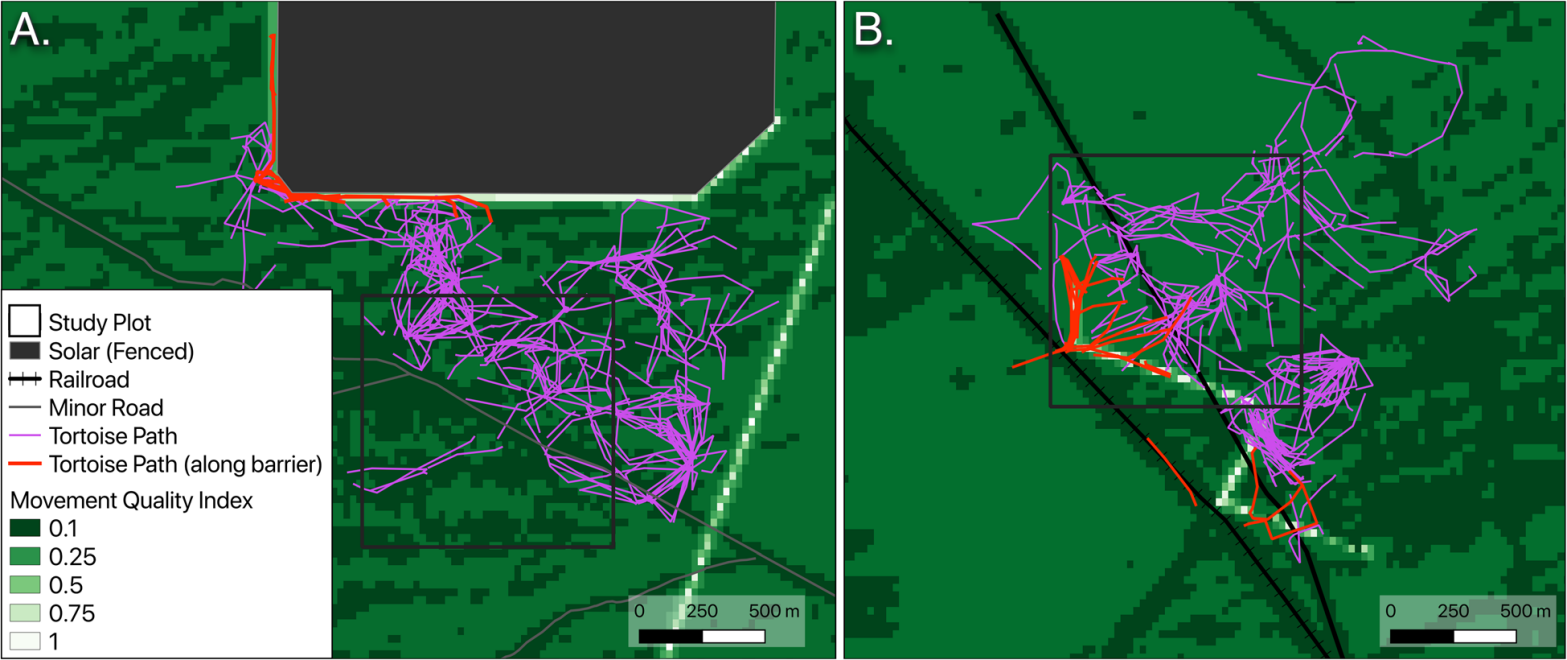
Tortoise movement quality maps accounting for selection of linear barriers in the area around the ISEGS South (**a**) and Nipton (**b**) study plots. Selection for fences (around solar and on bottom right) in the ISEGS South area and flood control berms in the Nipton area dilutes movement resistance from most landscape features. Tortoise paths (red lines) often follow these barriers, while paths away from the impassible barriers (purple lines) seldom occurred along roads

We found tortoise movement selection responded significantly to anthropogenic disturbances including roads, fences, flood control berms, and railroads (Fig. 4). The positive coefficient for distance to road indicates that tortoises are avoiding movement in proximity to roads. Despite this selection, 86% of tortoises that had a minor/dirt road within their estimated home range (*n* = 69) crossed it one or more times. The negative coefficients for distance to fence and flood control berms indicate that tortoises seem to be selecting for movement along these linear barriers. Although our results are less certain on the relationship of tortoise movement selection and railroads, the posterior suggests that the railroad functions in a similar fashion to the other barriers, but still overlaps zero. Of the seven tortoises that encountered railroad infrastructure, only two crossed (i.e. went under the railroad; no tortoises crossed over the rails) the railroad during our study period, one by crossing both times under the same culvert while the other crossed under a culvert, walked along the railroad and then crossed under another culvert over the span of 2 days. The tortoise that used the same culvert to cross both times spent several weeks on the other side of the railroad. The seven tortoises that encountered the flood control berm walked along the berm and ended their paths at the culvert (Fig. 5b). “Selection” for anthropogenic linear barriers (fences and the railroad) is likely a function of the propensity of tortoises to walk along barriers as they encounter them while trying to access areas on the other side, especially when the barrier prevents return to a prior known location.

### Movement quality predictions

**Fig. 6:**
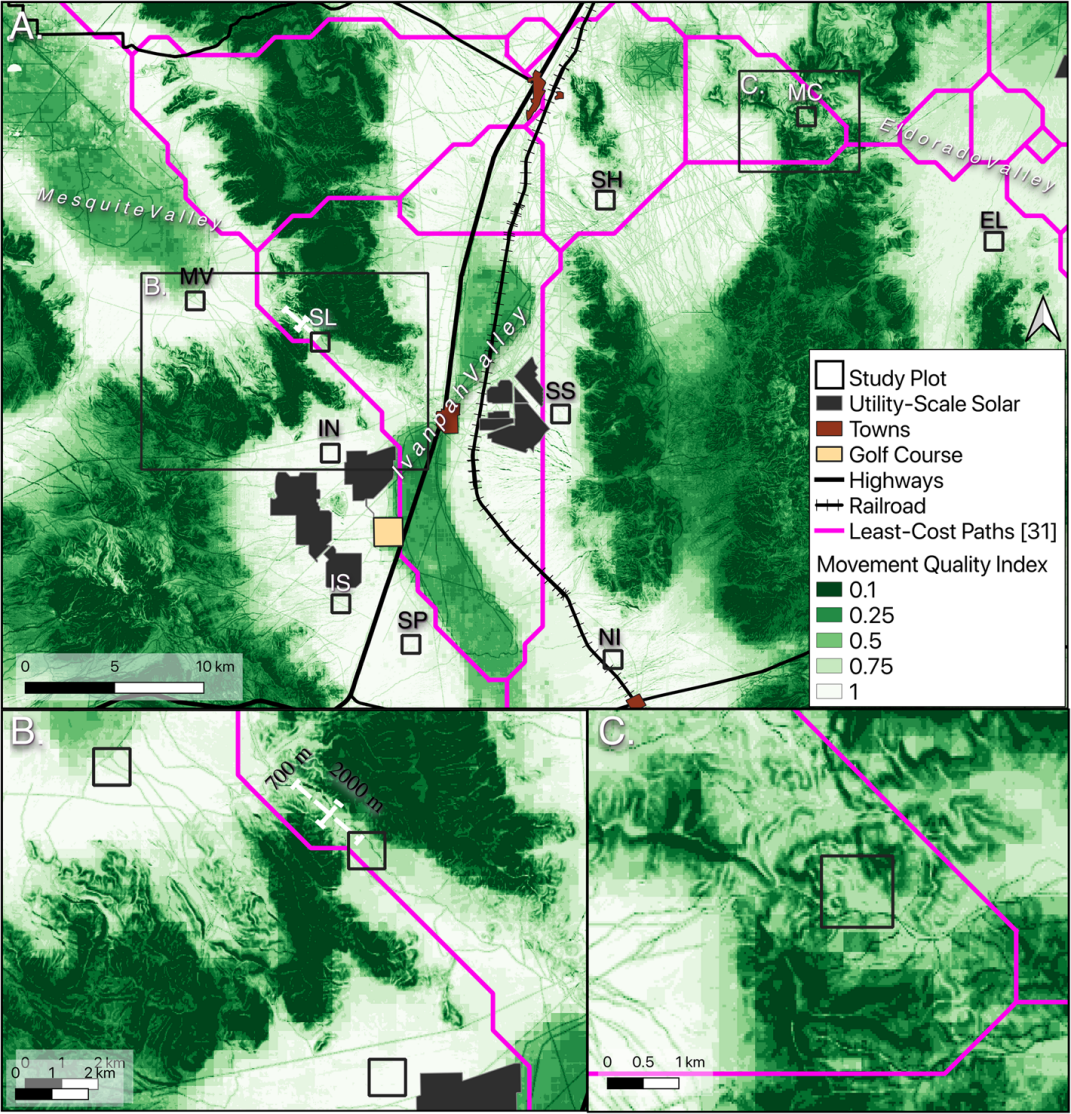
Tortoise movement quality map for entire study region (**a**), Stateline Pass area (**b**) and McCullough Pass area (**c**). Displayed values are quantiles of the movement prediction; higher values indicate areas that tortoises are predicted to move through with little resistance. Tortoises are less likely to move through rugged terrain and areas of low habitat suitability (dry lakes). The area to the northwest of the Stateline Pass study plot contains a strip of suitable movement habitat (represented by the white crosshairs) that functions as a corridor to connect the two valleys. In contrast, the area around the McCullough Pass plot contains terrain that is much more difficult for tortoises to traverse. Pink line overlays represent Least-Cost-Paths from [31]

Paths of tortoises that encountered anthropogenic linear barriers were often parallel to these barriers, and modeled selection for these features confounded other differences in movement quality (Fig. 5). After controlling for anthropogenic barrier selection, [84] predictions across the greater Ivanpah Valley area generally reflected tortoise habitat quality predictions, though differences between habitat and movement suitability in the areas of rugged terrain are evident (Fig. 6). To aid in visualization and interpretation, we classified our movement predictions into 10 quantiles, producing a map of “Movement Quality Index”. Closer examination of predictions within the two mountain passes, where we anticipated natural movement to be limited by terrain, show that the area northwest of the Stateline Pass plot contains the only corridor (roughly 700 m wide by 2000 m long) of high quality movement habitat between the Ivanpah and Mesquite Valleys (Fig. 6). In contrast, the area around McCullough Pass contains no obvious movement corridor—any tortoise attempting to move through the area would have to traverse areas of predicted low-quality movement habitat (Fig. 6). Overlaying the least-cost paths from [31] shows general concordance with our results, though several of the least-cost paths cross through areas where we predict low quality movement habitat, especially in mountainous terrain (Fig. 6).

## Discussion

Designing corridors to maintain connectivity for corridor-dwelling species requires careful consideration of both habitat and movement preferences [85]. In this study, we incorporated both movement selection and home range estimates to increase understanding of corridor functionality in a corridor-dwelling species. The two mountain passes in our study area offer insight into natural tortoise corridor functionality, in concordance with genetic connectivity between valley populations. Based on microsatellite data, the populations of tortoises in Mesquite and Ivanpah valleys historically maintained a high level of gene flow while the population within the McCullough pass area formed a unique cluster indicating a reduction in gene flow across the rugged terrain [26]. This aligns with the movement suitability we found in these areas—the Stateline Pass corridor is a nearly continuous strip (700 m wide and 2 km long) of high movement quality habitat between the adjacent valleys, while the McCullough Pass corridor has no areas of contiguous high movement quality habitat, requiring tortoises to move through considerable low quality movement habitat to disperse across the landscape. This distinction reinforces the importance of using movement data and finer scale raster resolution rather than modeled habitat suitability values to create resistance surfaces [13, 86, 87]. Use of the habitat suitability models previously developed for the Mojave desert tortoise at coarser resolution (1-km^2^ [25], 250-m^2^ [58]) suggests that resistance across Stateline and McCullough passes is roughly equivalent, while our movement-based model at a finer resolution (30-m) better represents the level at which tortoises are making movement decisions. Least-cost paths, built on habitat suitability and used to explain range-wide genetic distance in tortoise populations, suggest corridors through areas that cross areas we predict to be of low movement quality in our predictions ([31], Fig. 6), further reinforcing the need to consider the scale at which movement selection occurs in a species when determining corridors to maintain connectivity. If corridors to maintain historic connectivity were designated based on the predictions made from habitat suitability, important areas may not be included.

Our results provide similar connectivity predictions across the landscape to those of the Circuitscape model used in [52], though we predicted less movement of tortoises in mountainous areas, while the Circuitscape model suggests that high flow would occur over the smaller passes to the west of the Stateline Pass area. Although tortoises may be able to cross through the narrower locations, the available area in those passes is insufficient to accommodate multiple overlapping home ranges, and thus may not act as a functional corridor for generational movement of tortoises across the landscape. Despite male-male aggression during the breeding season, tortoises are not considered to be territorial and often have highly overlapping home ranges [88, 89]. Corridors of suitable movement habitat of this width (~ 700 m) may be wide enough to accommodate overlapping home ranges (a general guideline for corridor-dwellers outlined in [8]), though corridor-specific details, such as the composition of corridor edges (natural vs. man-made) and the length of the corridor may also play a role in functionality and effectiveness of mitigation actions. If a narrow corridor of usable movement habitat is short enough in length, it may still be effective for allowing tortoises to cross through areas of unsuitable movement habitat. However, if a corridor is longer, it may need to be wider to provide a functional corridor for tortoises. More research is needed to further determine the optimal ratio of length to width for corridors to preserve tortoise connectivity.

Estimated home ranges of tortoises at our study plots were generally larger than those reported in other studies [67, 88–90]. We present two hypotheses to explain this difference: we gathered finer scale data than other studies with our hourly GPS fix rate, and we used an autocorrelated kernel density estimator which accounts for spatiotemporal autocorrelation inherent with fine-scale telemetry data [91]. Gathering fine-scale data and using appropriate estimators are important in defining the home range of desert tortoise, and our results suggest that biweekly relocations may be sufficient to properly estimate home range size of desert tortoises [92]. A recent study has shown that the use of these estimators is important in properly determining home ranges in the closely related Sonoran desert tortoise [93] Our fine-scale data captures occasional tortoise forays (typically less than a week) outside of core use areas; tortoises that made these forays had large estimated home ranges. While we do not understand why tortoises make these movements, it is important to account for them when considering how much space tortoises need and highlights the utility of GPS loggers to collect movement data in this species. Our findings suggest that tortoises may have smaller home ranges in natural areas with features that restrict their movement (e.g. rugged terrain). However, smaller home ranges in mountain passes could also result from a higher availability of resources; rugged rocky landscapes have more opportunities for precipitation to pool for drinking, higher plant diversity, and lower temperatures that allow for longer periods of seasonal activity [68, 94].

Our fine-scale movement data for tortoises in mountain passes allowed parameterization of tortoise response to slope, a characteristic used in defining tortoise habitat, and one which has long been believed to restrict tortoise movement within rugged terrain [25, 90, 95]. Our model predicted a similar response to slope as assumed by the Gray et al. model [52], with movement habitat quality steeply falling as slope increased and predictions indicating little to no use of areas with greater than 30 degrees slope. Credible intervals for our slope selection coefficients were the only natural features that did not completely exclude zero, indicating that tortoises are not completely averse to moving through areas of higher slope, as indicated by the inclusion of higher slope areas within tortoise home ranges. Although our resolution is relatively fine (30-m), we may still be considering slope at too rough a scale with respect to tortoise movement; smaller features such as large boulders and small cliffs are not well represented at this resolution. Additionally other characteristics of sloped areas likely influence how tortoises move through them—resource (e.g. forage plants, thermal refugia) availability can also drive movement patterns of tortoises. Further research applying other methods of movement analysis should be used to better understand how tortoises modify movement pattern in areas of high slope; for instance, tortoises may make shorter movements in areas of higher slope and move perpendicular to the angle of the slope. Refining this pattern may require a shorter GPS fix rate, though location error inherent in GPS logger technology may ultimately prevent parametrization in lower available resolutions (10-m).

We found that tortoises select for areas of higher perennial vegetation cover and for areas that exhibit moderate wash characteristic intensity. Perennial shrubs are important cover for tortoises, providing areas for thermoregulation and protection from visual predators [56, 57]. Desert washes serve as important foraging habitat for tortoises [53], and tortoises often use burrows located in the banks of washes [96, 97]. Our finding that tortoises avoid large washes may be related to preference for higher perennial vegetation cover; large washes often have less shrub cover due to occasional flooding. Off-highway vehicle recreation, especially within washes, is associated with reduction of vegetation cover, tortoise mortality/reduced activity, burrow destruction, and soil compaction; washes with OHV activity had high wash characteristics in our wash layer [98, 99]. Although soil compaction may allow for easier movement, the loss of both forage and cover vegetation within these washes reduces the quality of these anthropogenically disturbed washes as tortoise movement habitat.

Roads have long been recognized as a threat to Mojave desert tortoise populations due to mortality risk and habitat degradation; tortoise densities are often lower near roads with considerable traffic due to increased mortality [35, 36, 38]. Our model indicates that tortoise movement habitat is degraded by minor roads; tortoises avoid moving through areas close to roads, though they are not completely averse to crossing them. It is not quite clear why tortoises choose to avoid moving near these low traffic roads—it may simply be a response to the absence of vegetation within roads (similar to the avoidance we found of large open washes), or it could be recognition of the danger of vehicular traffic. In addition to increasing perceived predation risk, reduction in vegetation cover alters thermoregulatory opportunities for tortoises resulting in a decreased thermal performance breadth [100]. Tortoises will congregate and burrow near roads to take advantage of higher vegetation growth associated with road runoff and pooling of precipitation on paved surfaces, though these activities are associated with less movement [36, 37, 101]. Several of our tortoises in the Nipton plot had home ranges that shared a border with the paved road (Fig. 6a), suggesting that the roadsides are suitable resident habitat, despite the road discouraging movement. Roadside patches of suitable habitat have been suggested as resident habitat for gopher tortoises and eastern box turtles, with animals showing avoidance of the road for movement yet carrying out typical habitat use patterns near the road [102, 103]. Further research should explore how desert tortoise movement is influenced by roads with different characteristics (traffic level, substrate, etc.), especially in areas where tortoises still have access to highways.

Despite the habitat fragmentation it causes, fencing between human development and tortoise habitat has become a common method to reduce human-tortoise conflict, especially with the intention of reducing mortality of tortoises from vehicle collisions [38]. Tortoises are often documented pacing back and forth along new fence construction within habitat, seemingly in an attempt to return to their prior home ranges to acquire known resources [104]. Movement along fences may expose tortoises to unsuitable environmental conditions and higher risk of predation [37, 105]. Many of the fences around the solar installations have been equipped with shade structures intended to reduce tortoise exposure to lethal temperatures, but it is not clear how these structures influence tortoise ecology or movement patterns. Other modifications of fences, such as adding angular protrusions from the fence, that deflect pacing tortoises away from the fence line and back out into the desert, could serve to mitigate some of the alterations to tortoise movement behavior, but their effects remain untested. Only one of the tortoises that encountered fences had a substantially smaller home range size than other tortoises, suggesting that the presence of fences does not typically reduce the amount of space a tortoise will use throughout the year. Another study nearby suggested that tortoise home ranges were reduced closer to newly constructed fences, though this finding was only significant for home ranges estimated with Minimum Convex Polygons, which provide inflated estimates of tortoise space use [37, 92]. Unfortunately, we do not have sufficient data to estimate the home ranges of the tortoises in our study prior to fence construction and cannot make inferences on how the addition of fences has altered individual tortoise behavior and space use. If the fences cause aberrant movement patterns and act as a hard edge to tortoise home ranges, we may expect to see a reduction in overall resource use and social interaction in affected tortoises; thus, long-term monitoring of these metrics is required to truly understand how these modifications influence individual fitness characteristics. The tradeoffs between the protections fencing provides relative to the potential disruptions to movement and behavior warrant further consideration.

Our results also indicate that tortoise movement is altered by flood control berms associated with railroad infrastructure in a similar manner as fences. The berms form linear barriers and were built to divert rare flood events underneath the railroad, but inadvertently influence tortoise movements by diverting their travel routes toward large underpasses. Railroads are barriers to the movement of gopher tortoises [40], and our limited data suggest a similar relationship between railroads and the Mojave desert tortoise. Tortoises that follow the berms often end travel at the entrance to the culvert, where they presumably shelter underneath the large rocks used to raise the railroad above the desert floor (only one tortoise has been observed to be doing this during VHF relocation events; all other data for tortoises in the vicinity of the culverts come from GPS loggers). Based on our data, railroad flood diversion berms do not result in tortoises pacing their perimeter such as we observed in relation to linear fences, which may be related to the shorter length of berms than fences, or to the berms being on the landscape for a much longer time (> 100 years) than the newly constructed fences. One tortoise has GPS points that suggest it climbed over the berm. Although we have described the railroad flood control berm mostly as a cost to tortoises, the few observations of culvert crossing we documented may actually increase connectivity among tortoises living on either side of the railroad and in that way provide a benefit relative to an absolute barrier that would exist from the railroad without them. One other study has shown that desert tortoises use culverts beneath transportation infrastructure during typical home range activity [106]. This suggests that directing dispersing tortoises to culverts through the use of linear barriers may increase the permeability of transportation infrastructure to connectivity of tortoise populations. The potential for barriers to be designed to act as positive landscape features for enhancing tortoise movement connectivity across anthropogenic barriers, such as fences that direct tortoises to culverts that provide passage under fenced highways, should be studied further.

We developed our model of tortoise movement using movements of adult tortoises within established home ranges. Recent studies have demonstrated that movements of animals during dispersal events may not be subject to same selection criteria as movements within home ranges. Rather, many species (including other tortoise species) often disperse through habitat unsuitable for long-term occupancy [18, 107–109]. Semi-aquatic amphibians will move through areas of unsuitable upland habitat (row crops) to disperse between wetlands [110]. Movement of kinkajous showed that although the species only uses forested areas for home ranges, dispersing individuals will cross land cover such as agriculture fields [111]. These differences in dispersal and within home range movements have been only been described for corridor-passage species; corridor-dwelling species may not follow a similar trend. Dispersal is still poorly understood within the *Gopherus* genus, and we did not have any tortoises with GPS loggers make large dispersal movements during the study period. While populations of the Mojave desert tortoise were historically continuously distributed across suitable habitat in the Mojave and Colorado deserts [31, 32], populations of the closely related Sonoran desert tortoise, *Gopherus morafkai*, are mostly confined to discrete foothill habitats [96, 112, 113]. Genetic analysis has shown that these patchy populations were historically connected by individuals dispersing through relatively unsuitable resident habitat in valleys; this behavior of dispersing through unsuitable resident habitat is not known in Mojave desert tortoise but could occur and play a role in maintaining genetic connectivity [108]. Several tortoises on our study plots, too small for GPS logger attachment, made dispersal movements that resulted in the establishment of new home ranges; generally these were large movements (> 1 km) across areas with high movement quality, with the exception of tortoises located in the McCullough Pass area (all dispersal movements [*n* = 4] < 1 km, Hromada et al. *unpublished data*). With continued monitoring of tortoises within these populations, we will be able to better describe how these different mountain passes influence dispersal patterns and the potential for tortoises to cross unsuitable habitat during dispersal movements.

Although our model was fitted using movement data from tortoises living within plots whose habitat characteristics varied considerably, we are limited in that we cannot observe or parameterize the response of tortoises to things they do not encounter (high slope, extreme high/low vegetation cover). Hence, our prediction of poor movement quality in areas such as the dry lakes in the Ivanpah Valley and areas of different vegetation communities, such as the blackbrush-Joshua tree scrub and pinyon-juniper woodlands present in higher elevations of our study area, are based on the positive linear relationship we found between tortoise habitat quality (based on climatic, physical and biotic factors) and tortoise movement propensity. While we do not have information on tortoises using all habitat types, we think our tortoises provide a reasonable sampling of the use of available habitat within this area, and their exclusion of using features (e.g. dry lakes and steep mountainous regions) is still valuable information toward evaluating their movement preferences. As the climate changes in the future, conditions for tortoise movement may be further degraded within the areas they currently inhabit while altering vegetation and climate conditions in higher elevations [114–116]. It is not well understood why tortoises do not typically use these other vegetation communities (e.g. sagebrush shrubland), though tortoises translocated into those vegetative communities made long movements in an attempt to return to familiar habitat conditions [47]. Future studies should work to understand how tortoises are using areas that represent ecotones between suitable and unsuitable vegetation communities, the propensity of tortoises to cross unsuitable habitat during dispersal movements, and how these movements play a role in shaping population connectivity over both ecological and evolutionary timescales. If climate change reduces suitable tortoise habitat to smaller patches of refugia as predicted in [115], dispersal movements through unsuitable resident habitat may become more important to maintaining population connectivity in this species.

Maintaining connectivity of animal movement through the conservation of corridors is crucial to maintaining tortoise populations across landscapes and assisting in the recovery of the species. Based on our results, the mitigation corridors left between utility scale solar (roughly 2 km wide) and the edge of tortoise habitat in the Ivanpah Valley (Fig. 1) are likely sufficient to maintain connectivity across the valley. This preservation of historical connectivity is conditional on retaining appropriate and intact habitat in those areas, as well as the rest of the valley remaining suitable habitat for tortoises and tortoise movements; further degradation may reduce connectivity and result in relatively isolated populations in less degraded areas. However, anthropogenic linear barriers at the edges of these corridors may alter corridor functionality. Within natural corridors formed by topographical features (Fig. 6b & c) movement is limited by topography along the edges yet this edge-of-corridor habitat is still suitable for other aspects of tortoise ecology (burrowing, foraging). This is in contrast to fencing, which alters tortoise movement behavior and provides little in the way of shelter or forage, potentially having negative fitness effects on resident tortoises [37]. Additionally, the presence of roads within suitable movement habitat potentially reduces the permeability of the landscape to tortoise movement which may alter long term landscape scale connectivity. Continued monitoring of the tortoises living alongside these human disturbances could ensure these mitigation corridors are functioning as intended over longer spans of time.

## Conclusions

We provide characterization of natural corridors for desert tortoise population connectivity using movement resistance and home range estimation. These natural corridors differ in general patterns of movement resistance, and tortoise home ranges are smaller in the mountain pass with more areas of higher movement resistance, indicating that these passes likely function differently in terms of movement connectivity. We also demonstrate that tortoise movement is influenced by anthropogenic disturbances—movement resistance increases in the presence of roads but decreases dramatically in the presence of hard barriers to tortoise movement. Mitigation corridors left on the landscape of our study area seem to be sufficiently wide to contain tortoise home ranges and allow for movement across the landscape, as long as habitat within them does not degrade in quality from further anthropogenic impacts. The use of fencing to create the edges of these mitigation corridors may have unintended consequences to individual tortoise fitness due to alteration of tortoise movements; continued monitoring could determine if this may undermine long-term connectivity and viability of tortoise populations.

## Data Availability

The datasets generated during and/or analyzed during the current study are not publicly available due the sensitivity of the species and easily accessible study sites but are available from the corresponding author upon reasonable request. Related data are available in [117].
